# Groundwater modelling for decision-support in practice: Insights from Sweden

**DOI:** 10.1007/s13280-024-02068-7

**Published:** 2024-10-14

**Authors:** Nikolas Benavides Höglund, Charlotte Sparrenbom, Roland Barthel, Emil Haraldsson

**Affiliations:** 1 Department of Geology, Lund University, Sölvegatan 12, 223 62 Lund, Sweden; 2 Department of Earth Sciences, University of Gothenburg, Box 460, 405 30 Gothenburg, Sweden; 3 Department of Biological and Environmental Sciences, University of Gothenburg, Medicinaregatan 7B, 413 90 Gothenburg, Sweden

**Keywords:** Data assimilation, Decision-support, Groundwater, Groundwater model, Uncertainty analysis

## Abstract

Groundwater is an essential resource for drinking water, food production, and industrial applications worldwide. Over-exploitation and pollution pose significant risks to groundwater sustainability. Groundwater models can be powerful tools for optimizing use, managing risks, and aiding decision-making. For this purpose, models should assimilate pertinent data and quantify uncertainties in outcomes. We examine applied modelling for characterization and decision support in Sweden from 2010 to 2023. We also review syllabi of water-related courses in Swedish higher education to assess the inclusion and extent of groundwater modelling education. We find that important academic advances in groundwater modelling over the past two decades have not translated into practical application within Sweden’s industry, that uncertainty quantification is rarely undertaken, and that groundwater modelling remains a low priority in higher education. Based on these findings, we offer recommendations that, while informed by the Swedish context, hold relevance for educational institutions, industry, and decision-makers internationally.

## Introduction

Groundwater constitutes approximately 99% of Earth’s liquid freshwater resources (UNESCO 2022). It plays a crucial role in the global water cycle and has a strong influence on surface water dynamics, contributing to streamflow and the biodiversity of riparian ecosystems (Land and Peters 2023). Globally, around half of the world’s population depend on groundwater as their primary source of drinking water, and 43% of water used for irrigation is sourced from groundwater worldwide (Siebert et al. 2010).

Potential threats facing the quality of groundwater include both anthropogenic and natural origins. Examples of anthropogenic threats are pesticides and fertilizers from agriculture, road salts from transportation networks, and spills or leakages of industrial contaminants or mine tailings that infiltrate the ground (e.g., Li et al. 2021; Abanyie et al. 2023). Natural threats include geogenic pollution that may result from the oxidation or reduction of certain minerals with a subsequent release of harmful elements (e.g., Kushawaha and Aithani 2021). Groundwater abstraction itself poses a multifaceted risk, including the acceleration of geogenic pollution by lowering the groundwater table, and potentially leading to saltwater intrusion in coastal areas (Fetter 2001). Additionally, the reduction in underground pore pressures can lead to land subsidence (Tzampoglou et al. 2023), posing a threat to the stability of structures. It can also alter the natural flow paths of groundwater near abstraction wells, which may cause solutes to move in unexpected directions. Ultimately, over-abstraction can deplete crucial groundwater resources which entire communities and ecosystems may rely upon (Jasechko et al. 2024), as well as contribute to a substantial part of the global sea-level rise (Wada et al. 2010).

Groundwater numerical models (henceforth referred to as groundwater models) can be powerful tools for managing groundwater resources, predicting the outcomes of various underground affecting actions, and supporting decision-making. They can be applied at varying spatial and temporal scales, and integrate ancillary processes of the hydrological cycle, such as evapotranspiration, water flow in the unsaturated zone, and groundwater–surface water interactions (Anderson et al. 2015). Arguably the most well-known code for simulating groundwater flow is MODFLOW (Langevin et al. 2017), developed by the US Geological Survey (USGS) in the 1980s and a public domain open-source software. MODFLOW, along with other simulation codes developed by both academia and private industry, typically work by dividing a study area into a mesh of smaller units (also referred to as ‘cells’) in which computation is undertaken. Each cell represents a portion of the subsurface at a specific location through the assignment of model parameter values that are representative of that location and scale. A model parameter is a variable internal to the model and its value can be estimated from data, such as hydraulic head, to reflect parameters of the physical system. Interaction between different parts of the hydrological cycle, as well as the model’s interaction with the surrounding areas and environment, is controlled through boundary conditions. Steady-state or transient groundwater flow is simulated within the model domain using either the finite difference or finite element method, depending on the software used.

Over the years, the term ‘groundwater modelling’ has evolved to encompass more than just forward simulation of groundwater flow. It often involves a wider range of processes within the workflow, aimed at enhancing the ability to make informed decisions on the effects of different actions or inaction concerning the groundwater system. These processes include data assimilation and uncertainty quantification (e.g., Anderson et al. 2015; Fletcher 2017; Doherty and Moore 2020). Data assimilation involves integrating observations from both soft data, such as expert knowledge, and hard data, such as field measurements, for example, by matching the model output to historical measurements (Fletcher 2017), a process often referred to as ‘history matching’ or ‘calibration’ (despite some differences, these terms are used interchangeably in this paper. Refer to the cited literature for term nuances). A modeller can history-match a model by manually adjusting the model’s parameters or by using calibration software, such as PEST (Parameter ESTimation; Doherty 2018), which has become an industry-standard software since its release to the public domain in the early 2000s. Calibration software generally works by iteratively seeking to improve the fit between model output and observations using, for example, the Gauss–Levenberg–Marquardt algorithm. Today, PEST is integrated in most commercial groundwater modelling software, and USGS has developed and maintains PEST++  (White et al. 2020), a similar, complementary software suite that shares its protocol with PEST.

Uncertainty analysis aims at quantifying the range of possible simulated outcomes, for example, by running the model multiple times using different plausible parameter combinations (often referred to as ‘parameter realizations’). Uncertainty in observations may be accounted for by generating different observation realizations, each differing slightly to account for measurement noise. Calibration-constrained uncertainty quantification attempts to reduce the uncertainty pertaining to the range of possible simulated outcomes, for example, by including history matching of each individual model realization. This ensures that the different simulated model outputs remain within an acceptable error margin from observed values (‘acceptable’ being a subjective term) thereby acknowledging what is uncertain, while simultaneously reducing the uncertainty by assimilating information in expert knowledge and historical measurements. Effectively quantifying (and preferably reducing) model uncertainty is essential for its role to serve as a decision-support tool (Doherty and Moore 2020), providing decision-makers with probabilistic assessments of simulated outcomes used for judging risks and possibilities.

Recently, a groundwater model became the focus of nationwide attention in Sweden after the Land and Environmental Court of Appeals (LECA) rejected the renewal of the permit application by Sweden’s largest cement producer, which included plans to increase the size of the company’s most productive quarry. Among the opponents motioning for the rejection were the Swedish Environmental Protection Agency (SEPA) and the County Administrative Board (CAB) of Gotland, as well as various interest groups (LECA 2021). Among the points of critique were inadequate model scale, insufficient calibration efforts (i.e., the model’s inability to match historical measurements), and a series of consecutive assumptions that, when combined, compounded the uncertainties, rendering the model unreliable (LECA 2021). LECA’s decision to reject the application, motivated by their critique aimed at the groundwater model, sparked what was referred to as the ‘cement crisis’ in Swedish mass media, potentially leading to the loss of up to 280 000 jobs and a monthly GDP decline of 0.74%, according to the Swedish Construction Federation (SCF 2021). The Swedish Geological Survey (SGU) was tasked to assess the consequences of ceased cement production at the site, finding that transitioning to alternative sites was infeasible (SGU 2021). Approximately one month after the rejection, the Supreme Court of Sweden (SCS) decided not to grant leave to appeal, thereby upholding the LECA’s ruling (SCS 2021). In reaction, the Swedish Parliament (SP) passed a legislative amendment to the Environmental Code, enabling a time-limited permit to be granted enabling the continuation of production at the site (SP 2021). Reviewed by the Council on Legislation (CL), the amendment was criticized for being unconstitutional as it tailored to the benefit of a single business operator (CL 2021).

This highly unusual situation provides an extreme example of the repercussions that can occur when applied groundwater modelling fails to meet the expectations of a ruling authority. And although it is the most publicly well-known case, it is not the only instance when the quality of groundwater models has been put in the spotlight. The design of groundwater models and their shortcomings in accounting for uncertainty have also been critiqued in other recent cases, including those heard by the Land and Environment Court (LEC) of Växjö (LEC 2021), as well as in several cases before the LECA (e.g., 2016, 2019, 2020, 2022, 2023a, b). Clearly, there is a need to review and improve the practical application of groundwater models within the industry, assess the points of critique, and implement standards grounded in scientific reasoning to enhance their reliability in decision-making processes. This study examines the application of groundwater modelling for characterization and decision-support in Sweden from 2010 to 2023, with our analysis reflecting key academic advances and the current state-of-the-art in the field. To bridge the gap between education and practical application, we also assess the syllabi of water-related STEM (science, technology, engineering and mathematics) courses in Swedish higher education institutions to identify the presence and scope of groundwater modelling education. Specifically, our goal is to:

- Identify the sectors in which groundwater models are applied and the objectives and predictions they are developed to assess.
- Assess the extent to which methods and tools outlined in academic literature have been adopted in Swedish decision-support modelling.
- Investigate the presence and scope of groundwater modelling in Swedish higher education institutions.
- Provide recommendations for future improvements for decision-makers, the industry, environmental authorities, and educational institutions.

Although the study’s focus is centered on the situation in Sweden, results and conclusions are highly relevant for the global groundwater community, in particular higher education institutions. Systematic studies seem to be missing, but anecdotal reference and comments published in editorials have been addressing a mismatch between the demand for hydrogeologists, the required skills and education offered, and a growing gap between science and practice worldwide (Simmons et al. 2012; Irvine 2018; Sowby and Walski 2021; Cherry 2023).

## Theoretical framework

This section presents the theory which underpins decision-support modelling and highlights recent advances at the forefront of the field. It also presents the context under which groundwater models are developed in Sweden.

### Groundwater model development and possibilities for decision-support

The properties of the underground are complex, with heterogeneities present at every scale. The disposition of the underground controls the movement and transport of groundwater and contaminants. Even with extensive field investigations, the volume of the underground that remains un-sampled outweighs the sampled volume by far. A model, typically developed using information contained in the sampled volume, is, by definition, a simplification of the system it is designed to represent. This inherent limitation is the ultimate reason for why the idea that a model can be (or should be) developed to replicate a natural system (or ‘reality’) is flawed (Doherty and Moore 2021). Rather, the design of a model should be guided by the specific prediction it is required to make (Ferré 2017; Guthke 2017; White 2017; Doherty and Moore 2021), facilitating the quantification of uncertainties pertaining to that prediction. This necessitates accounting for the fact that the disposition of the subsurface can be characterized only in a stochastic manner (Gómez-Hernández 2006; Doherty and Moore 2020).

For a model to ‘learn’ from historical measurements and express uncertainty, its input parameters must be allowed to vary. Therefore, the number of adjustable parameters and the manner in which they are assigned to the model and permitted to vary (i.e., the level of parameter complexity) directly influence the model’s capability to reduce and quantify uncertainty. The discussion on model complexity, including arguments both for simplicity and increased parameterization, is comprehensive (e.g., Hunt and Zheng 1999; Gómez-Hernández 2006; Hill 2006; Hunt et al. 2007; Guthke 2017; Doherty and Moore 2020, 2021; Hugman and Doherty 2022; Delottier et al. 2023), but ultimately favors higher levels of parameterization over the lumping of parameters. Parameterization by pilot points enables flexible, continuous boundary conditions and parameter fields, thereby allowing heterogeneity to emerge where necessary during history matching (de Marsily et al. 1984; Doherty 2003; White and Lavenue 2023). Although this approach traditionally results in increased model run time due to the larger number of parameters, it eliminates the need for the subjective guesses about partially known structural properties normally associated with the use of homogeneous zones. However, over the past two decades, significant advancements have been made in both enhancing the use of pilot points and reducing the increased run time associated with a larger number of parameters. These include the integration of geostatistical methods for guiding and constraining how parameters are assigned (Doherty et al. 2010), as well as adopting methods for reducing run time, including parallel and cloud computing technologies (e.g., Doherty et al. 2010; Fienen et al. 2011). More recently, the development of ensemble methods has decoupled the link between the number of parameters and model runs (Chen and Oliver 2013; White 2018; Alzraiee et al. 2022), enabling the use of highly complex models with thousands of parameters or more, while simultaneously providing predictive uncertainty quantification. For cases requiring exceptionally high model run efficiency, data space inversion using surrogate models offers a capable method for quantifying predictive uncertainty (Lima et al. 2020; Delottier et al. 2023), although at the cost of reduced parameter interpretability.

Modern decision-support software extends beyond just history matching. By utilizing the Jacobian matrix, which describes the relationship between observations and parameters, the value of past and future data can be explored (Dausman et al. 2010; Fienen et al. 2010). Data worth analyses can therefore guide future data collection efforts toward the most valuable types of data and identify the specific geographic locations where they should be collected (Kikuchi 2017). In cases where competing interests exist, such as achieving efficient contaminant removal while minimizing associated pumping costs, optimization software can be employed to explore various implementation strategies while taking uncertainty into consideration (White et al. 2022). In recent years, numerous open-source software packages have been developed that enhance productivity, transparency, and reproducibility (e.g., Bakker et al. 2016; White et al. 2016; Leaf and Fienen 2022), thereby facilitating more robust modelling workflows.

### Groundwater models in a Swedish context

Unlike some other countries, such as USA, Canada, Australia, and Denmark (Reilly and Harbaugh 2004; Wels et al. 2012; Barnett et al. 2012; Henriksen et al. 2017), Sweden does not have official groundwater model guidelines. However, a number of documents outline their applicability and usefulness in situations specific to contaminated sites and infrastructure projects. SEPA (2006) reviews alternative codes for simulating transport of solutes and provide recommendations for using models in risk assessments at contaminated sites. Trafikverket (the Swedish Transport Administration authority; 2013) provides guidelines for groundwater models specific to projects within the authority, and SGU (2019) provides a short overview of recommendations applicable over a wide range of projects. These documents, however, are comparably sparse in information pertaining to the concepts of data assimilation and uncertainty quantification, compared to, e.g., the guidelines outlined by Barnett et al. (2012).

**Fig. 1:**
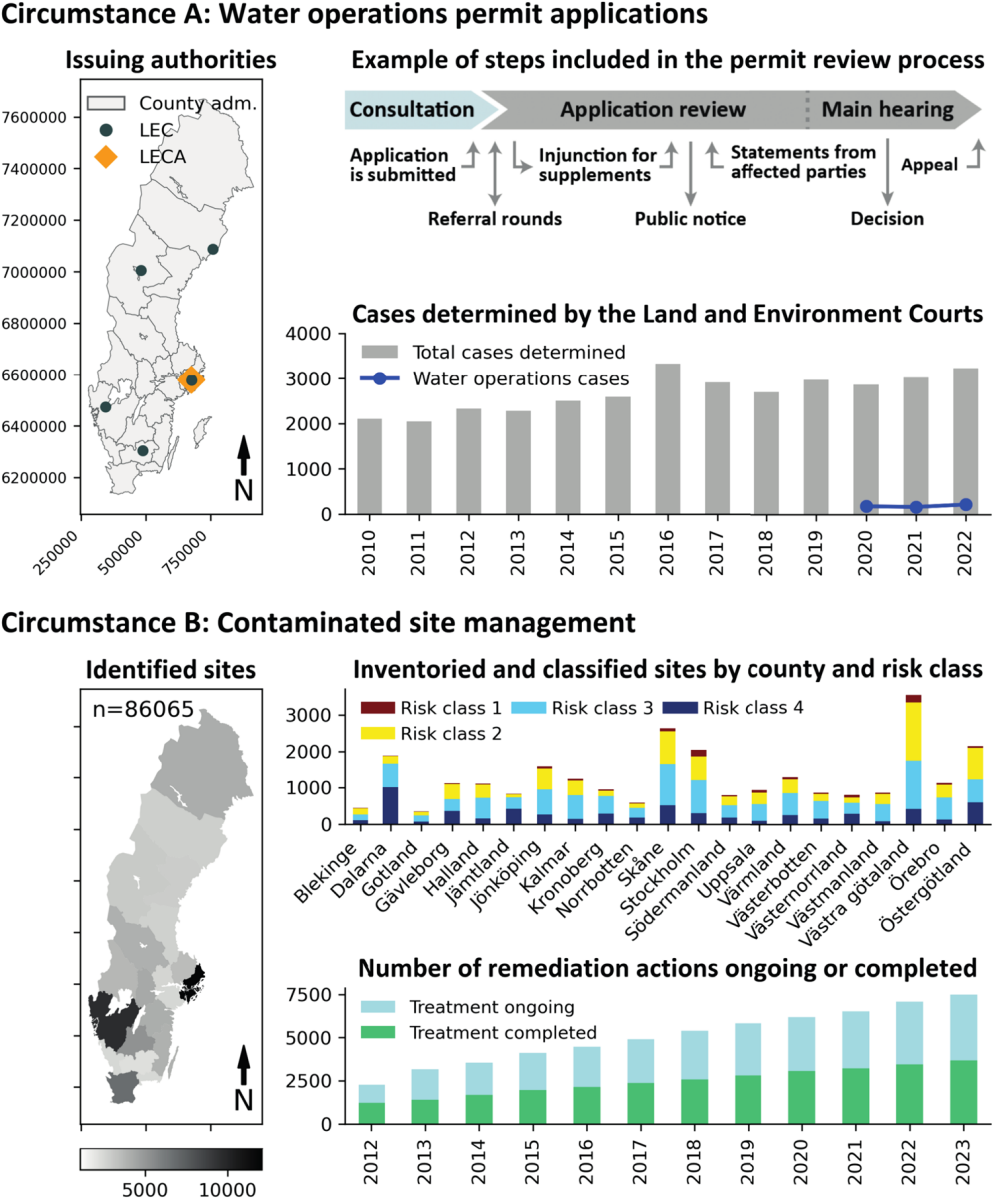
Illustration showing two common circumstances under which groundwater models are developed. Circumstance A outlines the permit application process for conducting water operations. The map shows the issuing authorities, including the CABs, LECs, and LECA. The flowchart illustrates the underlying steps within the permit application process. The bar plot shows the total number of cases determined by the LEC from 2010 to 2022 (Domstolsverket 2023), including water operations cases from 2020 to 2022 (SEPA 2023). Circumstance B illustrates identified, inventoried, and classified contaminated sites by county and risk class, including the cumulative number of remediation actions initiated or completed from 2012 to 2023 (SEPA 2024)

In Sweden, groundwater models are typically developed and applied within two main contexts: permit applications to the Swedish Environmental Code (SEC) and the investigation and remediation of contaminated sites. As shown (Fig. 1), the number of cases determined by the LEC and the number of initiated and concluded remediation treatments have steadily increased over the last decade. The SEC, which encompasses both of these circumstances, shares many principles with those of other nations belonging to the European Union and the OECD. These principles include the precautionary principle, which requires anyone conducting or intending to conduct an activity to acquire the necessary knowledge to prevent harm to human health and the environment, including the use of the best available technology not entailing excessive costs (BATNEC). Furthermore, the polluter pays principle mandates that the polluter should bear the cost of rectifying any environmental damage caused by their actions. The SEC further stipulates that anyone wishing to engage in water operations must first obtain a permit from the LEC or the CAB, ensuring the chosen site allows the activity’s purpose to be achieved with minimal intrusion and inconvenience to human health and the environment. The definition of water operations includes any action in a water area that aims to alter the water’s depth or location, including the abstraction or infiltration of groundwater.

SEPA has identified approximately 86 000 potentially contaminated sites in Sweden (SEPA 2024). Once identified, each site is prioritized based on its industry classification, a first general inventory, and a risk classification according to four levels, with 1 being the highest risk. For sites classified as level 1 through 3, a limited investigation is undertaken, which includes sampling to confirm the contaminants, and its severity (SEPA 1999). Included in the severity evaluation are assessments of the potential for contaminant transport. After the investigative phase, the preliminary risk classification is either confirmed or revised. Sites assigned a risk class of 1 or 2 will then be prioritized for more detailed investigation and remediation.

Within the two circumstances outlined above, there is ample room for utilizing groundwater models as a tool to provide decision-support in compliance with BATNEC and the SEC requirements.

## Materials and methods

Reports from 2010 to 2023 documenting the development and results of groundwater models in practical applications at sites in Sweden were collected by contacting the 21 County Administrative Boards and the 290 municipalities of Sweden, engaging with municipal water producers, industry professionals, and conducting searches on Google, Bing, and the DiVA portal (a Swedish digital archive and repository). The reason for selecting 2010 as a cutoff year is twofold: the requirement of a subjective decision to ensure the dataset reflects current practices; and, as described in section ‘Groundwater model development and possibilities for decision-support,’ numerous methods that facilitate robust modelling became available to a wider range of users outside of academia around this time, both in terms of software and computational parallelization capabilities. As the data collection proceeded, it became apparent that a significant number of reports were master’s theses written in collaboration with industry practitioners, often within actively ongoing projects. For this reason, we decided to include such theses in our collection of reports. A total of 121 reports were obtained, which were reduced to 101 after an initial screening. Reasons for filtering out reports include instances where a duplicate report was obtained from different sources, the report documented the use of an analytical rather than a numerical model, the report was written before 2010, or the study site was located outside of Sweden. Two of the reports encountered during our search for literature documented discrete fracture network (DFN) models developed to assess long-term conditions affecting the spread of nuclear waste in deep storage within crystalline bedrock. Because their unique characteristics compared to the rest of the dataset, particularly in terms of modelling approaches, focus on research and method development, and distinct geological settings, these cases were omitted from this study. As several authors requested anonymity for their clients’ sake, the decision was made to anonymize all reports by means of removing the author’s name, organization, document title, and the approximate site coordinates from the database.

A spreadsheet was created and populated with qualitative, quantitative, and Boolean data extracted from the reports. Qualitative data include information on the type of report (e.g., technical report or thesis), sectors (e.g., infrastructure or water security), objectives of model assessment, simulation software used, temporal discretization (steady state or transient), parameterization approaches, and calibration methods. The ‘water security’ category pertains to both water production and well-head protection. Quantitative data include the publication year, approximate model coordinates, the number of observations and model parameters, the number of words describing the model setup, and the number of words presenting and discussing the model results. The rationale for including the number of words discussing the setup and results is to provide a rough estimate of the transparency and reproducibility of the model workflow’s (as indicated by the setup description) and interpretability (as reflected in the discussion of model results), thereby offering insight into the level of understanding a decision maker would derive from these reports. In most cases, the number of model parameters was not explicitly stated in the report. For this reason, they had to be estimated based on the description and figures of the model setup, as documented in the reports. This was not the case for observations, however, which were often provided or could be counted from figures. For clarity, note that the number of model parameters counted in our dataset pertains to both fixed and adjustable parameters. In the groundwater modelling literature, ‘the number of parameters’ often refers to the number of parameters adjusted during calibration. This topic is further elaborated in the discussion section. Boolean data include whether calibration, sensitivity analysis, and uncertainty quantification were performed or not.

Syllabi for water-related STEM courses were downloaded from the websites of Swedish higher education institutions. After filtering out courses that were no longer actively offered (but still remained in the course catalogs), 165 courses remained. A spreadsheet was created and populated with qualitative, quantitative, and Boolean data. Qualitative data include the name of the educational institution, department, course title, and course code. Quantitative data include course credits, which translate to course length (in weeks). Boolean data indicate whether water, groundwater, groundwater modelling, calibration, sensitivity analysis, and uncertainty analysis were discussed in the course syllabus.

## Results

**Fig. 2:**
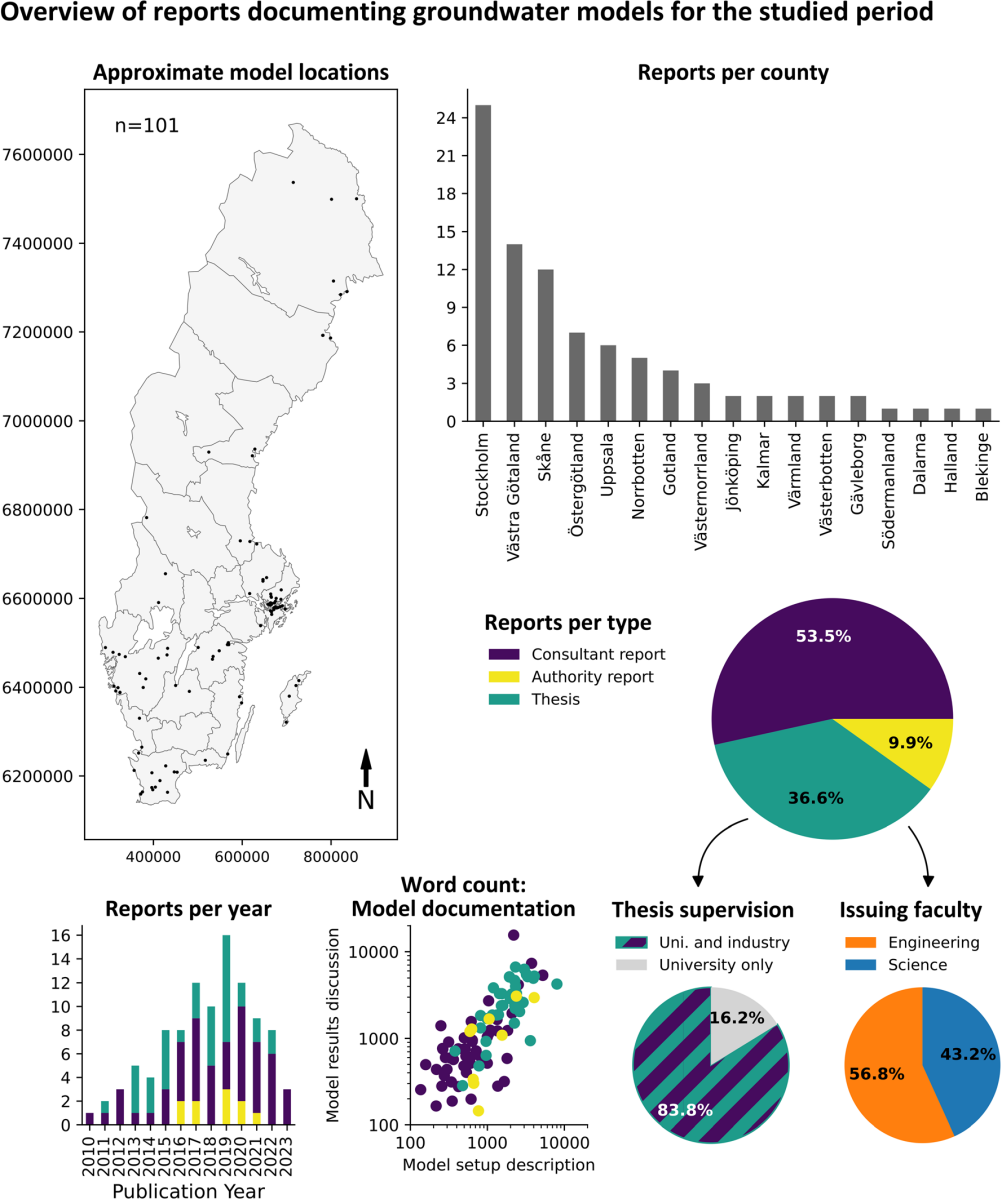
Overview of the main dataset analyzed in this study. The map shows the approximate geographic location the model was developed for. Remaining plots show the number of documented models per county, report type, publication year, and approximate level of reproducibility and interpretability, as indicated by the number of words describing the model setup and results

The geographic distribution of model locations (Fig. 2) shows that most models were developed to assess groundwater-related issues in southern Sweden, with just over half of the reports documenting models located in the three most populous counties: Stockholm, Västra Götaland, and Skåne. Model locations in northern Sweden are concentrated in the coastal cities of Sundsvall, Skellefteå, and Luleå, as well as inland regions where mining is prominent. Consultant reports (also referred to as technical reports) comprise 53.4% of the documentation, followed by master’s theses (36.6%) and authority reports (9.9%; Fig. 2, lower right). Of all the master’s theses analyzed, 31 (83.8%) were written in collaboration with a consultancy. Master’s students from both engineering and science faculties were represented, with slightly more reports written by engineering students (56.8%) than by science students (43.2%). The number of model reports published per year increased between 2010 and 2019, to decline thereafter (Fig. 2). Generally, the word counts for the setup descriptions and discussions of model results were higher in master’s theses compared to consultant reports (Fig. 2), except for a small number of consultant reports that were even more comprehensively described.

**Fig. 3:**
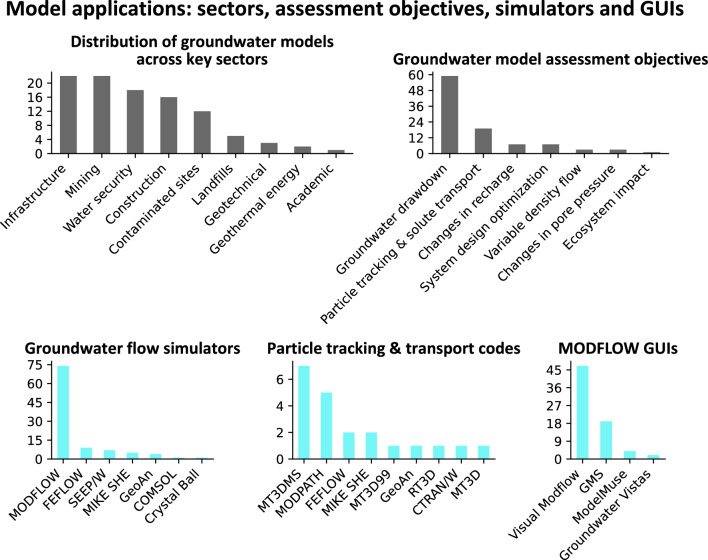
Bar plots showing the number of groundwater models documented within the model reports dataset and their distribution across key sectors, the objectives they were developed to assess, and which simulators were used within their workflows

Among the various sectors (Fig. 3) for which the models were developed, infrastructure and mining were the categories with the most models developed (22 each). Subcategories of infrastructure-related projects include tunnel, bridge, road, and railroad construction. The subcategories of mining include the mining of ballast, ore, and carbonates. Water security (drinking water production and protection; 18 models) includes municipal and private water supply, as well as managed aquifer recharge aimed at increasing freshwater production and suppressing saltwater intrusion. Construction (16 models) mainly includes urban development, while geotechnical (three models) concerns ground stability. The remainder of the categories (contaminated sites, landfills, and geothermal energy; 12, 5, and 2 models, respectively) are self-explanatory. One report, a master’s thesis not written in collaboration with industry professionals, did not easily fit into the other categories and was therefore labeled as academic.

Although most models were developed to assess more than one objective, the primary objective of most models (59 models; Fig. 3) was to quantify groundwater drawdown. Particle tracking and solute transport (flow paths and contaminant fate analyses) were the primary objectives of 19 models. Changes in recharge and system design optimization, addressed by seven models each, involve quantifying the effects of different future climate change scenarios on groundwater resources and optimizing remediation designs, which include flow barriers and drainage systems. Assessments of variable density flow (three models) include the effects of thermal and saline variability on aquifers. Changes in pore pressure, addressed by three models, involve quantifying the effects of changing pressures on the stability of slopes and structures. Only a single model was designed with the direct primary objective of assessing ecosystem impact.

Most of the groundwater simulation codes used were MODFLOW (> 70%; Fig. 3), followed by alternative industry-standard software including FEFLOW, SEEP/W, MIKE SHE, and COMSOL. The majority of the particle tracking and transport codes were also associated with MODFLOW simulators, including MT3DMS, MODPATH, MT3D99, RT3D, and MT3D. The most used graphical user interfaces (GUIs) were Visual MODFLOW (47 models), followed by GMS (19 models), ModelMuse (4 models), and Groundwater Vistas (two models).

**Fig. 4:**
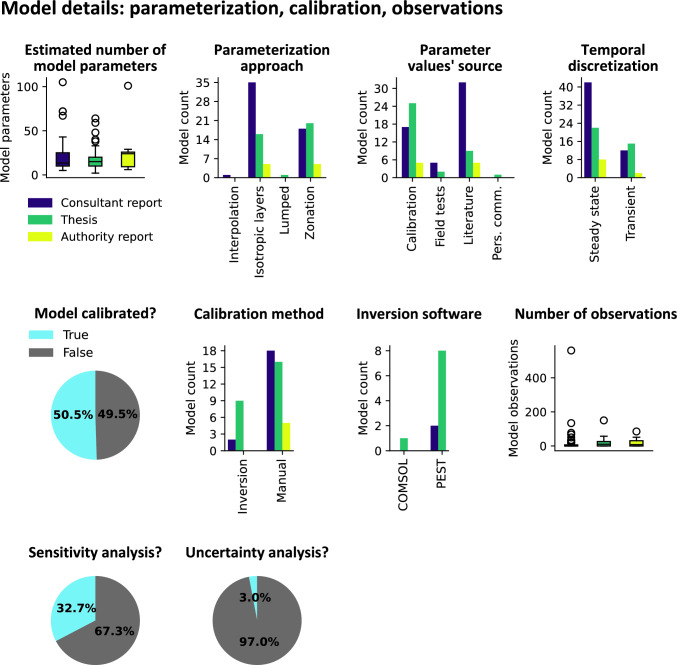
Box plots and bar plots showing details pertaining to model parameterization and calibration per report type. Pie charts showing Boolean data indicating the percentage of which models underwent calibration, sensitivity analysis, and uncertainty quantification

The estimated number of model parameters as counted from figures, tables, and documentation is generally below 25, with outliers for some models reaching slightly over 100 parameters (Fig. 4). The most common parameterization approach involves the use of homogeneous isotropic layers, followed by zonation. In models featuring units with low hydraulic conductivity, such as clay, vertical anisotropy was sometimes introduced at a factor of ten lower than the horizontal conductivity. Models developed by consultants are nearly twice as likely to utilize homogeneous layers than zonation, whereas master’s students show a slight preference for zonation over layers. Only two models employed alternative parameterization approaches, involving two contrasting methods: interpolation of parameter values and a lumped parameter approach. None of the models utilized pilot points. The most common methods for determining parameter values involved using literature-derived values and model calibration. For models developed by consultants, using literature values was nearly twice as common as calibration. For models developed as part of a master’s project, calibration was more than twice as common compared to sourcing values from the literature. A few models (< 7%) were parameterized by translating parameter values derived from the analytical solution of field tests onto isotropic layers. A single model was parameterized with values derived through personal communication. Most models (> 65%) were developed to simulate groundwater flow under steady-state conditions, compared to transient conditions, especially in models developed by consultants.

Just over half of the models underwent some form of calibration (Fig. 4) with manual calibration (also known as ‘trial-and-error’; > 78%) being the most common calibration method. All of the models calibrated using inversion software, except for one, were calibrated using PEST. The majority of models utilized fewer than 35 observations (i.e., calibration targets), with a few models (< 25% of calibrated models; 12 models) exceeding that number. One model utilized as many as 560 observations. Parameter sensitivities were explored in approximately a third of all models (Fig. 4), and uncertainty quantification for assessment objectives was conducted in 3% of all models.

**Fig. 5:**
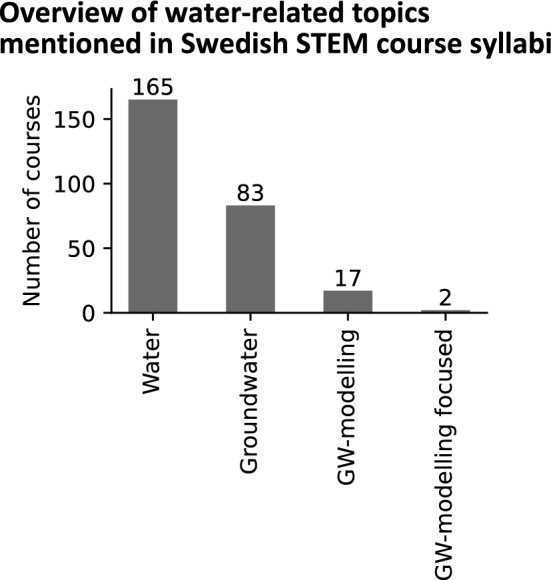
Bar plots showing the distribution of groundwater-related terms mentioned in the syllabi of STEM courses in Swedish higher education

Out of the 165 water-related STEM courses for which syllabi were downloaded and inventoried, groundwater was discussed in approximately half of them (Fig. 5). Groundwater modelling was mentioned as a part of the curriculum in 17 of the syllabi, and two courses were specifically focused on groundwater modelling: one course spanning five weeks and the other seven weeks. Given that the seven-week course included both surface water modelling and groundwater modelling, it is estimated that roughly half of this course’s duration was devoted to groundwater modelling. One of these courses mentioned calibration in its syllabus. Understanding the concept of model uncertainty was mentioned as a goal in four of the syllabi, although these courses, except for one, were primarily focused on surface water modelling. None of the syllabi specifically mentioned data assimilation. The average duration of courses that mentioned groundwater modelling as part of the syllabus was 5.8 weeks.

## Discussion

The discussion is structured as follows: First, we will discuss the datasets, including potential biases and their implications. Second, we will examine our findings concerning the syllabi of STEM courses in our higher education institutions. We consider this examination important for framing the subsequent discussion on the results of the model reports with background insights into groundwater education. We will then discuss our findings pertaining to the model reports, and the role of decision makers and incentive structures. Lastly, we will provide suggestions for improvements.

### Representativeness of the datasets

In the data collection phase, we found groundwater-based water producers to be reluctant to share their groundwater model reports, as they contained classified information about the locations of production wells. Although not all contacted producers reported using groundwater models, including the unshared models in our dataset would have increased representation in the ‘water security’ category. Some of the industry professionals were also reluctant to share their work, out of respect for their clients. Had they been shared with us, the distribution of report types would have shifted toward a higher percentage of consultant reports. How significant this shift would have been, we cannot say.

It is important to note that water permit applications, to which many of the consultant reports contribute, are considered open data and often become searchable over time as search engines index them. In practice, however, municipal, regional, and judicial repositories, where such reports are filed, are difficult to search and lack transparency. Often, they are searchable only by date, case number, and case title. Within such a case, numerous reports could be filed, of which a report documenting a groundwater model could possibly exist. Upon contact with several municipalities, it was indicated that searching for such documents for which this study examines, specific case numbers were required. This led to a paradoxical situation where finding the information needed requires prior knowledge of specific details, significantly limiting our ability to search these repositories. Thanks to the willingness of officials to inquire among themselves on our behalf, we were nevertheless provided with numerous reports despite this challenge. It is likely that the number of reports, and similarly the distribution of consultant reports, would have increased if these repositories had been more transparent and easier to access. The apparent decline in published reports after 2019 likely stems from a combination of a time lag in the indexing of these reports, as they have to circulate and eventually be stored on a publicly accessible repository for search engines to locate them, and a decline in modelling-related theses, which has been anecdotally observed among some educators. Including additional reports would also likely reshape the dataset in ways that cannot be foreseen at this point.

Concerning the dataset covering syllabi of water-related STEM courses, it should be noted that the information presenting the course content is relatively brief, often less than a page, and does not provide extensive details on concepts and methods taught during the course. By comparison, this dataset represents a relatively small portion of the information analyzed in this study. For a more thorough understanding of how extensively certain topics are covered, we recommend conducting more detailed investigations that involve contacting course coordinators and surveying for in-depth course content. We encourage researchers in future studies to delve deeper into groundwater-related education, as groundwater (including groundwater modelling) is a complex topic that warrants further exploration.

Despite the potential limitations discussed above, we consider our findings significant, clear, and relevant to the field. They will now be discussed in detail below.

### Groundwater modelling in higher education

**Fig. 6:**
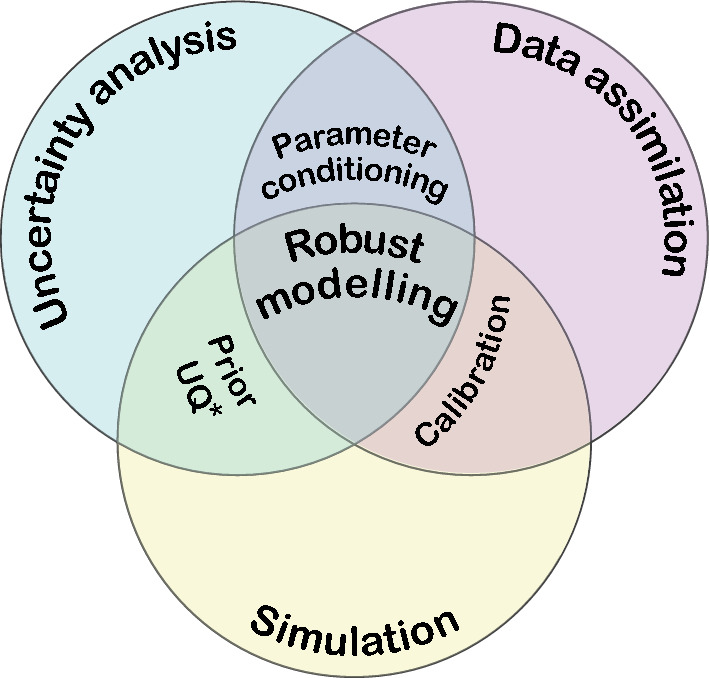
Venn diagram showing important components in groundwater modelling. *Prior uncertainty quantification, as demonstrated by Hemmings et al. (2020), may in certain cases provide a sufficient approach without the involvement of history matching. Many models encountered during our search for literature place solely within the simulation category

We observe that courses mentioning groundwater modelling in their syllabi tend to focus on forward simulation of groundwater flow. However, as presented in the theoretical framework of this paper, groundwater modelling also encompasses other important concepts that bring significance to the forward simulation, including data assimilation and uncertainty quantification (Fig. 6). Without incorporating these concepts, the practical value of forward simulation becomes questionable, as it renders the model unable to learn from data and blind to uncertainty. Teaching students the concepts and methods of data assimilation and uncertainty quantification, we argue, is more common in adjacent fields of study within the hydrological cycle, such as in meteorology. This should also be a priority within groundwater education, as these skills contribute to robust modelling workflows and are transferable to related fields of study. This approach would provide students, especially those intending to focus on modelling later in their careers, with a solid foundation for conducting sound scientific analysis.

Groundwater is our most abundant liquid freshwater resource. All its nuances cannot be covered in a single course. As Simmons et al. (2012) have observed, many university curricula have struggled to incorporate advances in hydrogeology for decades. In a more recent editorial, Cherry (2023) argues that a lack of space for groundwater courses in the curriculum, as well as a lack of teachers specializing in groundwater, hinders the development of future experts in the field; expertise that will be critical in addressing the expanding global water crisis. For these reasons, it is essential for higher education leadership to recognize this and understand the need for greater emphasis on covering various aspects of groundwater, thereby increasing the likelihood of its responsible management.

### Applied groundwater modelling in Sweden

Similar to our findings on groundwater modelling education, our analysis of the model reports’ dataset confirms a gap in the integration of robust data assimilation strategies within workflows. Although we acknowledge such workflows could be documented in technical reports elsewhere, as shown (Fig. 4), none of the workflows analyzed in our study included the use of pilot points or other highly parameterized approaches. Instead, most models were configured to use homogeneous layers and zones as their primary parameterization approach, resulting in most cases in the use of fewer than 25 model parameters. It should be noted that in models that underwent calibration, the majority were history matched using far fewer parameters (often fewer than ten) through manual adjustments of parameter values. In models that underwent calibration using inversion software, authors often reported that a subsequent manual calibration provided a better fit with measured data. We suggest that this is because the parameterization approach used by the model developers is too rigid and simplistic, not allowing for the required parametric heterogeneity to be expressed during history matching. Even when parameter values were permitted to vary within reasonable limits, we often observed a single parameter value applied to every cell in a layer, rendering the inversion software ineffective in its application.

In some reports, we observe instances of vagueness, confusion of concepts, and questionable practice. Examples of vagueness include the idea that the metric for which to judge a groundwater model is how well the model resembles reality, or claims like ‘the best model is the simplest model that achieves the desired outcome.’ It is mentioned that conservative parameter values are used, without stating the values used or how they were assigned, or that calibration is redundant due to input parameters already being reasonable. Confusion of concepts includes claims that a model is calibrated after applying parameter values sourced from literature, analytical methods or even conceptual interpretation (calibration is the process of adjusting parameter values in order to match historical measurements with model output), or that a model is validated even though residuals exceed several meters (a groundwater model cannot be validated, it can only be invalidated; Bredehoeft and Konikow 2012). Such statements suggest that the authors might not be fully aware of methods to quantify uncertainty or assimilate data, or that models were developed and tested with insufficient resources for applying these approaches. This could also indicate that in some cases, objectives and principles of modelling are not well understood. In a few exceptional cases, we found reports where calibration targets were removed due to high residuals that could not be decreased during calibration. Such actions raise concerns about transparency in the modelling process and risk preventing important discussions on potential measures for addressing model deficiencies.

Notably, only 3% of model workflows included some form of uncertainty analysis, among which were documented in one thesis and two consultant reports from large infrastructure projects. This is significant, as it implies that the majority of decisions, which are supported by these models, are made without any knowledge about their reliability or lack thereof. To put it clearly, the average groundwater model tends to overlook uncertainty and is hampered in its ability to learn from data. This disconnection between approaches in groundwater research on the one hand, and in practical application on the other, has been identified by many authors before us (e.g., Irvine 2018; Sowby and Walski 2021; and references therein) in contexts that extend far beyond Sweden. However, this issue can be addressed, as state-of-the-art open-source software for robust modelling is available, as presented in the theoretical framework section.

### Decision makers and incentive structures

The prevalent gap in addressing model uncertainty and improving robustness of workflows outlined above highlights an opportunity for significant improvement. However, the challenge exists not only in the educational or technical domain but also within the frameworks that guide decision-making processes. The current praxis for issuing water operations permits may be reinforcing the current situation. Recent analyses reveal a first instance permit approval ratio of 85% by the LECs and 80% by the CABs, respectively (SEPA 2023). Applications denied in the first instance may be submitted for appeal and retried at a higher level. Several legitimate concerns regarding the quality of models, raised by SEPA and various interest groups during consultation and feedback in the application reviews (see the introduction, Fig. 1, and cited cases in the LECs and LECAs), underscore the need for scrutiny. As observed in some reports, authors state that model development follows standard practices. SGU, the authority responsible for the national environmental quality goal ‘Good Quality Groundwater,’ frequently provides expertise on groundwater models during permit reviews. However, it is observed that SGU’s recommendations for groundwater model development (SGU 2019), while providing valuable insights and being among the few sources available, rely on traditional perspectives that may not fully encompass recent advancements in data assimilation and uncertainty quantification. The incentives for initiating change in current praxis are sparse when models are developed according to existing practices and if permits are granted with little regard for the often significant uncertainties in hydrogeology, whether these uncertainties are illuminated through uncertainty analysis or not. By omitting uncertainties and robust practices used in academia for many years, the precautionary principle and the principle of BATNEC, as stated in the Environmental Code, is not being adhered to, elevating the risk of an application being rejected. The costs for resubmitting and appealing a water permit denial can be many times more costly than performing the model work with best available techniques, highlighting uncertainties. Although this may constitute a form of financial incentive, it is likely ineffective in this regard due to important details of modelling practices not being well understood within the practice of environmental courts.

### Suggestions for future improvement

Education is the foundation from which practice evolves. Several authors have previously identified gaps between groundwater research, education, and industry, attributing this to limited research exposure (e.g., Sowby and Walski 2021) and stagnation of university curricula despite significant advancements over past decades (e.g., Simmons et al. 2012). For a model to effectively serve as a decision-support tool, data assimilation and uncertainty quantification are essential processes that bring meaning to simulations within a modelling workflow. Given this premise, we would from our perspective propose a hypothetical course focused on groundwater modelling, to span 10 weeks, a standard duration equivalent to half a semester. This course would aim to adopt a holistic approach to groundwater modelling, grounded in decision-support modelling theory as outlined in the theoretical framework and the literature cited in this paper. To facilitate efficient supervision, reduce costs, and accommodate fluctuating student numbers over the years without relying on costly licenses, adopting a blended learning approach (Dziuban et al. 2018) is recommended. This approach incorporates modern industry-standard open-source software and interactive learning platforms, such as digital notebooks, drawing inspiration from the Groundwater Modelling Decision Support Initiative (GMDSI; Hugman et al. 2022). This approach would liberate classroom time for teacher–student interactions and allow for in-depth discussions, enhancing the learning experience with exercise materials that students engage with independently. Reflecting the critical role of groundwater in society and addressing the gap in groundwater-focused education within higher education systems, this course could serve as an important component of a hypothetical master’s program in hydrogeology, aligning with Cherry’s (2023) perspective that groundwater education is essential to combat the global water crisis.

Industry professionals operate in a highly competitive setting. Those willing to adopt the methods outlined in the theoretical framework and the literature cited within this paper can gain a competitive advantage as they build their skills. Additionally, they will be able to identify potential workflow issues early, offering the opportunity to address them proactively. This not only makes their modelling workflows more robust but also prepares them to meet the standards of increasing demands by issuing authorities. We recommend several recent case studies that implement robust workflows for addressing groundwater-related issues commonly observed across the globe. These include probabilistic capture zone analyses for well-head protection (Fienen et al. 2022), assessing the risk of streamflow depletion due to groundwater abstraction (White et al. 2021), identifying benefits and limitations of managed aquifer recharge (Standen et al. 2022), finding the optimal trade-off between pumping and the risk of well salinization in coastal aquifers (Coulon et al. 2024), probabilistic contaminant source identification (Hugman et al. 2023), exploring strategies for optimizing remediation system design (Fienen et al. 2024), characterizing surface water-groundwater exchange for identifying discharge of polluted groundwater (Höglund et al. 2023) among many others. The commercial GUIs already used by many industry professionals, to varying degree, support the adoption of sophisticated data assimilation strategies, including uncertainty quantification, making the transition toward robust modelling partially a question of using new workflows within familiar software. However, for access to state-of-the-art capabilities in model partner software, which allows complete customization to suit each site’s unique conditions, we recommend that practitioners refer to GMDSI (gmdsi.org) for a repository of worked examples, videos, webinars, and monographs on the topic.

We distinguish between decision-makers involved in permit approval processes, and those concerned with more direct issues related to the management of specific groundwater systems. Guidelines provide decision-makers of the first category with a framework for making consistent decisions regarding the use and protection of groundwater resources. Their implementation in some countries (e.g., USA, Canada, Australia, and Denmark) likely reflects their leadership in groundwater modelling and reliance on groundwater for freshwater. Although the implementation of guidelines can increase the quality of modelling workflows and related reports, their implementation may also bring negative side-effects (e.g., Kamali Maskooni et al. 2024). Due to their potential rigidity and requirement for adherence, they may drive cost and hamper the adoption of new and improved methods as they evolve, eventually hindering the use of groundwater models rather than facilitating it. We propose a system of guideline recommendations that permit deviation when necessary, allowing for flexibility and facilitating innovation. These recommendations can be based upon principles for sound modelling (Fig. 6) and be developed as a ‘living document’ in collaboration between representatives of an expert authority (such as the Geological Survey), academia, and the industry. Such guideline recommendations can equip decision-makers of the first category with a robust foundation on which they can decide to rule in favor of a permit or against it when a model does not live up to the recommendations. It will also increase the incentives among practitioners to pursue more robust practices, enable modellers to clearly understand certain expectations, and allow deviations from the recommendations when a justifiable motivation is presented. Consequently, this will likely lower the risk of permit denial caused by misunderstandings among stakeholders, as demonstrated by LECA (2021). The design and refinement of these guidelines should be prioritized for future research, considering their role in enhancing decision-making processes and the need to balance rigor with flexibility. This also points out that knowledge on modelling among the staff at environmental authorities need to improve. We further recommend that decision-makers in the first category carefully consider concerns raised by authorities, as well as those of local interest groups, as they often possess local expertise concerning geographic areas that may be impacted by groundwater abstraction and other related activities. We also want to clearly point out that a groundwater (or environmental) model can, if designed to do so, quantify the impact, including related uncertainties, of groundwater affecting actions on adjacent ecosystems like streams or wetlands. This implies that in many cases where concerns about such impacts are raised, a model designed for this purpose could assess them directly, rather than inferring the impact based on the effects of an adjacent system, as was observed in several reports in this study.

We encourage decision-makers in the second category, usually stakeholders seeking to apply for water operations permits, to incorporate numerical modelling into their decision-making frameworks earlier within projects and more frequently. It is our observation that a norm exists within the industry, suggesting that numerical models should primarily be used in large, costly, and complex projects, and after analytical models have proven insufficient. We disagree with this norm. Hemmings et al. (2020) showed that early uncertainty quantification in predictive models can improve decision-making and potentially reduce costs. It is our opinion that there is a trend in applications for water operations permits to often aim at demonstrating minimal environmental impact and a low risk of exceeding environmental quality standards. The inadequate modelling efforts and the resulting unreliable models could, if not a major change among the modelling community, lead to more environmental court rejections and cause an increased cost for society, and those who applies, i.e., the costumers of the modelling consultants. By adopting an outcome-agnostic approach to modelling, and by utilizing optimization codes to explore optimal solutions in cases where competing interests exist, the chance of obtaining permit approval may actually increase, due to the demonstrated rigor of the work. We encourage the view of modelling as an ongoing process, rather than as a finite product, and to see it as a tool that can be shaped for the assessment of specific questions and updated as new information is collected. Finally, we recommend that decision-makers from both categories inquire about the uncertainty of model results, including an overview of how uncertainties were assessed.

## Conclusions

In Sweden, most groundwater models are developed to assess groundwater-related issues in infrastructure, mining, water security, and construction. Geographically, the models are concentrated to the three most populous areas: Stockholm, Västra Götaland, and Skåne county. The main objectives the models were set to assess include the evaluation of groundwater drawdown and determination of flow paths and velocities. MODFLOW, employed via well-known commercial GUIs, is the most commonly used code for groundwater simulation. Most models were developed by consultants, but a significant number of models were also developed as part of master’s projects in collaboration between universities and consultants. The models were generally developed to represent local geology using homogeneous layers and zones and were designed to represent groundwater flow under steady-state conditions. Around half of the models underwent some form of calibration, with a strong preference for ‘trial-and-error’-calibration. Generally, the models utilized few parameters extrapolated over large areas, which often made it difficult to fit simulated values to historical measurements, and notably, only 3% of models underwent some form of uncertainty quantification. The low prevalence of robust data assimilation and uncertainty quantification strategies in practical applications reflects the limited focus on these concepts in current groundwater education. In the previous section, we presented suggestions for higher education institutions, the industry, and decision-makers. Although based on a Swedish context, they can likely, partially or fully, be transferable to contexts within other countries. They are briefly summarized as follows:

- On the premise that groundwater is a fundamental resource to society, education in groundwater modelling necessitates a full-length course dedicated to the subject, rather than as a supplement to a broader course in hydrogeology. This ensures that students, who will play pivotal roles in its management, are equipped to recognize and quantify uncertainties related to model outcomes.
- In applied groundwater modelling, emphasis tends to fall on simulation, overlooking the important concepts of data assimilation and uncertainty quantification. Recognizing and incorporating these concepts into both industry practices and higher education is crucial for improvement.
- Implementing guideline recommendations that emphasize the integration of simulation, data assimilation, and uncertainty quantification, yet are flexible enough to allow for deviation from these recommendations when justified, can promote robustness of model workflows, reduce misunderstandings between stakeholders and decision-makers, as well as facilitate the adaptation of new methods as they develop.
- Innovative approaches grounded in robust modelling principles are plentiful in recent literature. We have suggested specific papers pertaining to often encountered situations that industry practitioners may derive inspiration from.

Lastly, considering the number of models encountered during our search for literature, the number of cases processed within the LECs, as well as the number of contaminated site remediation projects initiated each year, we suggest there is significant opportunity to enhance decision-making outcomes through the wider use of models grounded in robust modelling principles.
